# Association of Stool Frequency and Consistency with the Risk of All-Cause and Cause-Specific Mortality among U.S. Adults: Results from NHANES 2005–2010

**DOI:** 10.3390/healthcare11010029

**Published:** 2022-12-22

**Authors:** Xinwei Peng, Jibin Li, Yuwan Wu, Hongji Dai, Henry S. Lynn, Xi Zhang

**Affiliations:** 1 Department of Biostatistics, Key Laboratory on Public Health Safety of the Ministry of Education, School of Public Health, Fudan University, Shanghai 200032, China; 2 Department of Clinical Research, State Key Laboratory of Oncology in South China, Collaborative Innovation Center for Cancer Medicine, Sun Yat-sen University Cancer Center, Guangzhou 510060, China; 3 Department of Pediatrics, Xinhua Hospital, Shanghai Jiao Tong University School of Medicine, Shanghai 200092, China; 4 Department of Epidemiology and Biostatistics, Tianjin Medical University Cancer Institute and Hospital, National Clinical Research Center for Cancer, Tianjin Key Laboratory of Cancer Prevention and Therapy, Tianjin’s Clinical Research Center for Cancer, Key Laboratory of Molecular Cancer Epidemiology of Tianjin, Tianjin 300060, China; 5 Clinical Research Unit, Xinhua Hospital, Shanghai Jiao Tong University School of Medicine, Shanghai 200092, China

**Keywords:** stool consistency, bowel movements, mortality, cancer mortality, CVD mortality

## Abstract

Background: Prior studies on the relationship between bowel health and mortality have generally focused on the individual association of stool frequency or consistency with mortality but did not present a joint association. Therefore, we aimed to systematically evaluate the individual and joint associations of stool frequency and consistency with all-cause and cause-specific mortality in this study. Methods: A total of 14,574 participants from the National Health and Nutrition Examination Survey 2005–2010 were incorporated in this analysis. Survey sample-weighted Cox proportional hazards models adjusted for potential confounders were used to estimate hazard ratios (HRs) between bowel health measures and mortality risks. Results: During a median of 7.6 years of follow-up, 1502 deaths occurred, including 357 cancer deaths and 284 cardiovascular disease (CVD) deaths. The bowel habit of the most participants was 7 times/week (50.7%), and the most common type was “Like a sausage or snake, smooth and soft” (51.8%). Stool frequency displayed a parabolic relationship with all-cause mortality, and less than 7 times/week is a significant risk factor for mortality (HR for 1 time/week: 1.43, *p*-values = 0.04. HR for 6 times/week: 1.05, *p*-value = 0.03). Analyzing the joint association of stool frequency and consistency on mortality clarified the limitations of only inspecting the effects of either individual factor. Compared with 7 times/week of normal stool, infrequent soft stools at 4 times/week were associated with 1.78-, 2.42-, and 2.27-times higher risks of all-cause, cancer, and CVD mortality, respectively. Conclusion: Analyses of bowel health should consider the joint effects of stool frequency and stool consistency. Self-appraisal of stool frequency and consistency may be a simple but useful tool for informing about major chronic illnesses.

## 1. Introduction

Stool frequency and consistency are well-known and easily acquired indicators for bowel health and gut microbiota [1]. There is a dynamic balance between the microbiota and the host [2]. Cumulative evidence indicates that human gut microbiota plays an important role in holistic health and disease progression by regulating metabolic function and interacting with the immune system [3,4,5,6,7]. Dysbiosis of gut microbiota can often be directly reflected in abnormal stool status [8]. Previous studies have reported that gut microbiota dysbiosis increased the risk of gastrointestinal disorders, obesity, cardiovascular diseases (CVDs), allergy, neurological disorders, and cancers [1,2,5,9,10,11,12,13,14]. On the other hand, both stool frequency [15,16] and consistency [17] have been shown to correlate with colonic transit time, which is an indicator of bacterial metabolism and mucosal turnover in the gut. Moreover, stool consistency is closely correlated with major microbiome markers [17] and compositional changes in gut microbiota [18,19]. Thus, stool frequency and consistency have been used as indicators of whole-gut transit time and to monitor changes in intestinal health [20].

Several previous studies have reported associations between stool frequency with all-cause and CVD-specific mortality [21,22,23,24] but some of the results have been conflicting possibly because of the different cutoffs used for categorizing stool frequency. Other studies have focused on symptoms of constipation or diarrhea but did not examine in detail the relationship between stool frequency and mortality [22,23,24,25]. Stool consistency has been included in the diagnostic criteria for chronic constipation and diarrhea in both the Rome III criteria [25] and the Bristol stool form scale [20], but no human study has focused on the relationship between stool consistency and long-term mortality. In general, little is known about the individual and joint association of stool frequency and consistency with mortality, especially with cancer mortality. Therefore, this study aims to systematically evaluate the individual and joint associations of stool frequency and consistency with all-cause and cause-specific mortality using data representative of the U.S. adult population.

## 2. Methods

### 2.1. Study Design and Population

**Figure 1:**
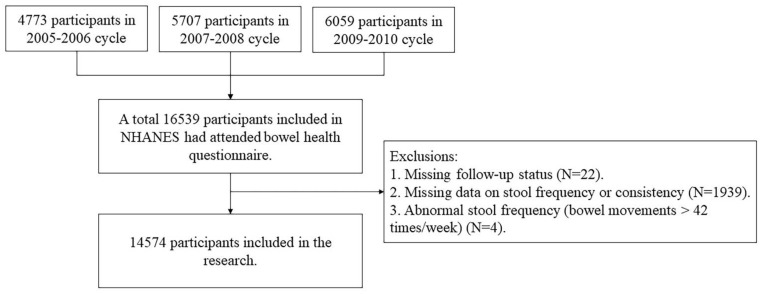
Flowchart of participants included and excluded in the final analysis (N = 14,574).

We used the 2005 to 2010 data from the National Health and Nutrition Examination Survey (NHANES). NHANES is a stratified, multi-stage, probability cluster sampling survey to evaluate the health and nutritional status in the U.S. general population, and its study design has been described in detail [26]. We combined the data from three consecutive surveys, including 4773 participants from 2005–2006, 5707 participants from 2007–2008, and 6059 participants from 2009–2010. Participants with missing follow-up status, missing bowel health data or abnormal stool frequency were excluded (N = 1965). Altogether, 14,574 participants were included in the analyses (Figure 1).

The ethical approval was given by the National Center for Health Statistics Ethics Review Board (https://www.cdc.gov/nchs/nhanes/irba98.htm (accessed on 20 September 2020)) and all survey participants provided informed written consent.

### 2.2. Definition of Exposure

NHANES participants were asked “how many times per week do you usually have a bowel movement?”. Their reported stool frequency was analyzed as a quantitative variable, and as an ordinal variable with five categories: 1~2 times per week, 3~6 times per week, 7 times per week (reference group), 8–21 times per week, and more than 21 times per week, in accordance with prior studies [27,28,29] and the distribution of the data.

Stool consistency was self-reported according to the Bristol stool form scale, which asked participants to select their “usual or most common stool type” by viewing the images and descriptions of seven stool types: separate hard lumps, like nuts (type 1); like a sausage but lumpy (type 2); a sausage shape with cracks in the surface (type 3); like a sausage or snake, smooth and soft (type 4, defined as the reference group); soft blobs with clear-cut edges (type 5); fluffy pieces with ragged edges, a mushy stool (type 6); and watery, no solid pieces (type 7). In accordance with prior analyses [20], stool consistency was examined in its original scale and as a trichotomous variable with 3 types (soft: types 5–7, normal: types 3 and 4, hard: types 1 and 2).

### 2.3. Definition of Outcome

The outcomes included all-cause mortality, cancer-specific mortality, and CVD-specific mortality. Deaths were confirmed by linking with the National Death Index public access files that were available by the end of 31 December 2016. Causes of deaths were classified as cancer (C00-C97) or CVD (I00-I69) using the tenth version of the International Classification of Disease (ICD-10). Survival time was calculated as the time interval between the date of study entry and date of death or 31 December 2016, whichever occurred earlier.

### 2.4. Definition of Covariates

Baseline information on sociodemographic characteristics, lifestyle habits, personal health status, physical examinations, and medical history were collected. The general information on age, sex, race/ethnicity (non-Hispanic white, non-Hispanic black, Mexican American, others), alcohol consumption (current drinker or non-current drinker), smoking status (never, former, and current), physical activity (low or never, moderate, or vigorous), dietary fiber intake (g/day) [18,30,31], annual family income (0–$19,999, $20,000–$34,999, $35,000–$74,999, or $75,000 and over), and history of chronic diseases (i.e., diabetes, cardiovascular disease, or hypertension) were self-reported by participants according to the standard questionnaires. The body measurement data, including weight and height, were collected by trained health technicians, who were accompanied by a recorder during the examination. Body mass index (BMI, kg/m^2^) was calculated by dividing the body weight (kg) to the squared the height (m).

### 2.5. Statistical Analysis

The bivariate associations between stool consistency and the 5-category stool frequency variable and other categorical baseline covariates were examined by chi-square tests, while differences in age between stool consistency types and stool frequency were compared by one-way ANOVA. The chi-square tests and ANOVA were weighted using sampling weights supplied by NHANES to allow for inference back to the U.S. general adult population [32].

Survey sample weighted Cox proportional hazard models were used to evaluate the associations between bowel health and the risk of mortality in terms of hazard ratios (HRs) with accompanying 95% confidence intervals (CIs). All models were adjusted for age, sex, and race/ethnicity, BMI, smoking status, alcohol consumption, dietary fiber intake, recreational physical activity, annual family income, and history of chronic diseases. The analyses first examined the separate effects of stool frequency and stool consistency on mortality, and then their joint effects on mortality. We explored possible nonlinear associations between stool frequency and mortality by modeling stool frequency using a restricted cubic spline polynomial with 3 knots at 4, 7, and 14 times/week. When examining the joint association of stool frequency and consistency on mortality, the 7 types of stool consistency were grouped into 3 types (soft: types 5–7, normal: types 3 and 4, hard: types 1 and 2) in accordance with prior analyses [20] and to avoid data sparseness that may lead to poorly fit models.

Sensitivity analyses were conducted to investigate the robustness of the results after excluding (1) patients with severity incontinence (Fecal Incontinence Severity Index scores > 30) since previous evidence suggested that the quality of life among these participants might be significantly impaired [33]; and (2) participants with a history of chronic diseases in order to minimize the potential for reversed causality. Additional sensitivity analyses examined whether consideration of competing risk would alter the conclusions of the survival models on cause-specific mortality.

All statistical analyses were performed using SAS Software (SAS 9.4, Inc., Cary, NC, USA), and a two-sided *p* < 0.05 was considered statistically significant.

## 3. Results

### 3.1. Characteristics of Participants

The mean age among 14,574 participants was 46.7 years (range: 20–85 years), and 51.1% of whom were female. The majority (76.5%) had normal stool consistency (Table 1). Younger Black females who were non-smokers, non-drinkers, with low BMI or low dietary fiber intake were more likely to report having hard stools (Table 1) and bowel movements of 1–2 times/week (Table 2). In contrast, participants who reported soft stools or bowel movements of more than 21 times/week, tended to be older, with high BMI, and current smokers.

After a median follow-up of 7.6 years (range: 10.0 months–11.0 years), a total of 1502 deaths (10.3%) occurred, with 284 deaths due to CVD (18.9%) and 357 deaths caused by cancer (23.8%).

### 3.2. Stool Frequency and Mortality

**Figure 2:**
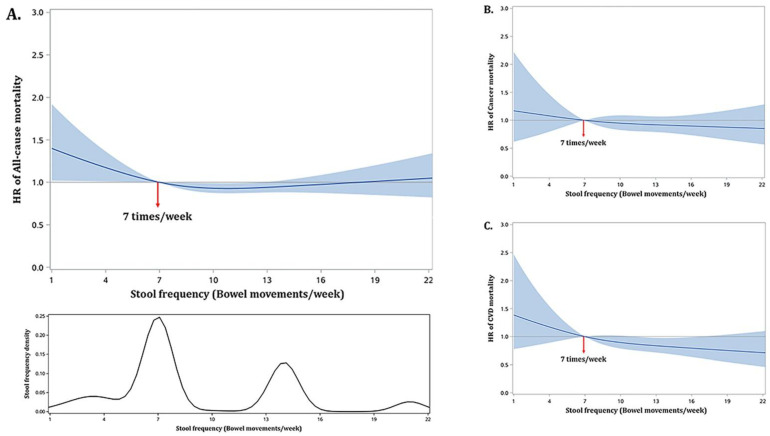
Non-linear relationship between stool frequency and all-cause (**A**), cancer- (**B**), and CVD-specific mortality (**C**) in U.S. adults (NHANES 2005–2010). The non-linear relationship is depicted by using a restricted cubic spline with 3 knots at stool frequencies of 4, 7 (reference), and 14 times/week of bowel movements. The solid curves represent HRs estimated by using weighted Cox regression models adjusting for age, sex, race/ethnicity, family income, BMI, smoking status, alcohol consumptions, physical activities, dietary fiber intake, and history of chronic diseases. The shaded area represents the 95% confidence intervals of the HRs.

Stool frequency displayed a parabolic relationship with all-cause mortality where the lowest risk occurred at 10 times/week (adjusted HR: 0.94; 95% CI: 0.89, 0.99; *p*-value = 0.02) when compared to a stool frequency of 7 times/week (Figure 2A). Higher risks were observed for lower frequencies with an adjusted HR of 1.05 (*p*-value = 0.03) at 6 times/week increasing to an adjusted HR of 1.43 (*p*-value = 0.04) at 1 time/week, while slightly lower risks were observed for frequencies from 8 to 12 times/week (*p*-values < 0.05). The risks of cancer mortality and CVD mortality decreased with increasing stool frequencies (Figure 2B,C). The hazard of cancer mortality at 7 times/week was, however, not significantly different from the hazards at other frequencies (*p*-values > 0.25), while the hazard of CVD mortality at 7 times/week was significantly higher than the hazards at frequencies of 11 to 16 times/week with adjusted HRs ranging from 1.15 to 1.25 (*p*-values < 0.05).

### 3.3. Stool Consistency and Mortality

Compared to participants with sausage-smooth stools, those with stools resembling soft-blobs with clear-cut edges showed a significantly higher risk of all-cause mortality (adjusted HR: 1.48; 95% CI: 1.16, 1.88; *p*-value = 0.002) and cancer mortality (adjusted HR: 1.60; 95% CI: 1.01, 2.53; *p*-value = 0.04) (Table 3). When the 7 stool types were further grouped into hard, normal, and soft types, participants with hard stools (adjusted HR: 1.24; 95% CI: 1.04, 1.48; *p*-value = 0.02) and those with soft stools (adjusted HR: 1.31; 95% CI: 1.09, 1.59; *p*-value = 0.005) showed higher risks of all-cause mortality compared to participants with normal stools. Participants with soft stools also displayed a higher risk of cancer mortality (adjusted HR: 1.36; 95% CI: 0.96, 1.92) compared to those with normal stools, but the result was not statistically significant (*p*-value = 0.09).

### 3.4. Joint Associations

**Figure 3:**
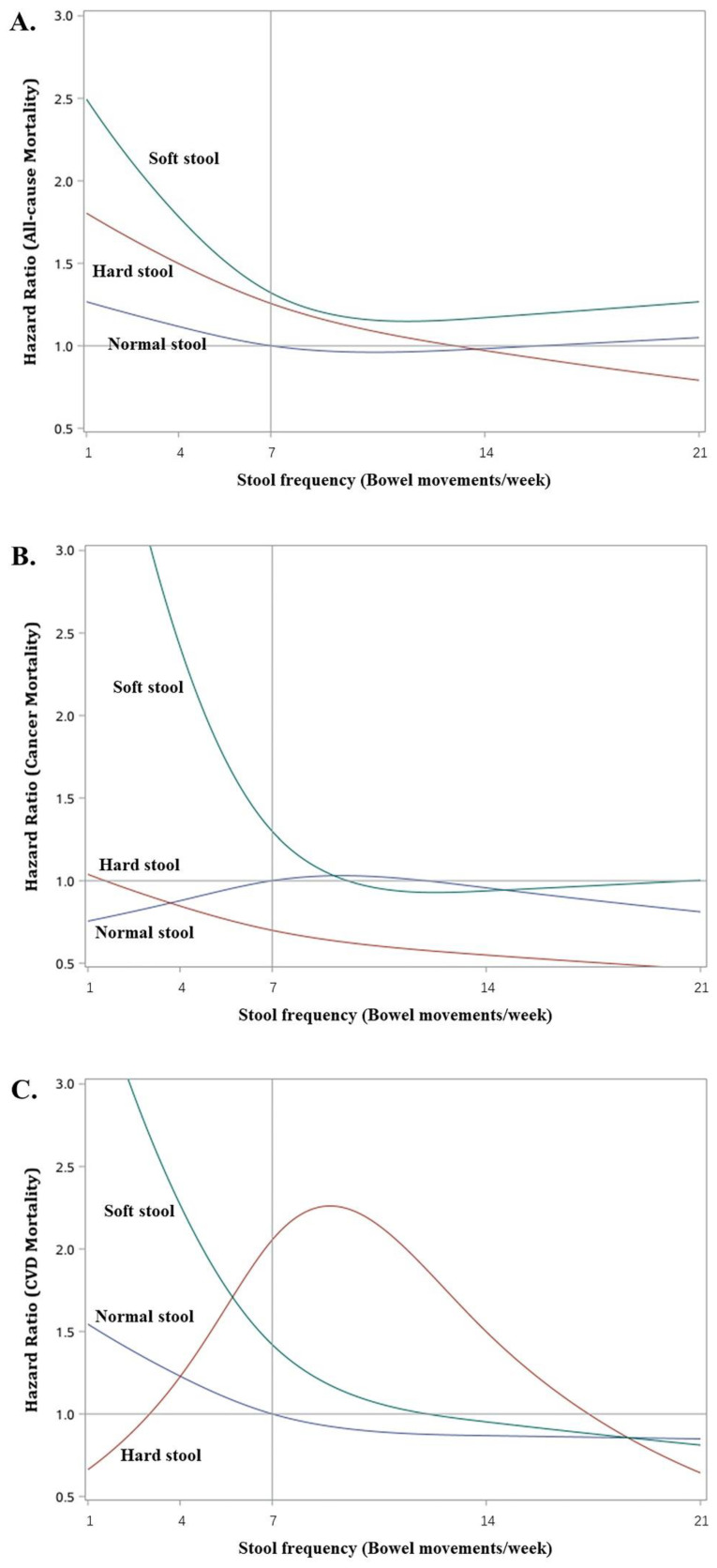
Non-linear relationship between stool frequency and all-cause mortality (**A**), cancer mortality (**B**), and CVD mortality (**C**) stratified by stool type in U.S. adults (NHANES 2005–2010). The non-linear relationship is depicted by using a restricted cubic spline with 3 knots at stool frequencies of 4, 7, and 14 times/week. The solid curves represent HRs (with respect to normal stool at 7 times/week) estimated by using weighted Cox regression models adjusted for age, sex, race/ethnicity, family income, BMI, smoking status, alcohol consumptions, physical activities, dietary fiber intake, and history of chronic diseases. Normal stool includes type 3 (a sausage shape with cracks in the surface) and type 4 (like a sausage or snake, smooth and soft) stools; Hard stool include type 1 (separate hard lumps, like nuts) and type 2 (like a sausage but lumpy) stools; Soft stool include type 5 (soft blobs with clear-cut edges), type 6 (fluffy pieces with ragged edges, a mushy stool) and type 7 (watery, no solid pieces) stools.

Figure 3A–C plot the adjusted hazard ratios (with respect to normal stool type at 7 times/week) at increasing stool frequencies for all-cause, cancer, and CVD mortalities. Participants with soft stools had L-shaped frequency–mortality relationships in all three figures, although the magnitudes of the increase in hazards at low stool frequencies (<7 times/week) were different depending on the mortality outcome. For example, at 4 times/week the adjusted HR was 1.78 (95% CI: 1.17, 2.73, *p* = 0.0076) for all-cause mortality, while they were 2.42 (95% CI: 1.31, 4.47, *p* = 0.0048) and 2.27 (95% CI: 1.12, 4.60. *p* = 0.0231) for cancer and CVD mortality, respectively (Table 4). Among participants with normal stool, the changes in hazards at different stool frequencies were much milder, and there were no statistically significant adjusted HRs (all *p*-values > 0.5) for all three mortality outcomes. Participants with hard stools had elevated risks of all-cause mortality at low stool frequencies; e.g., adjusted HR = 1.50 (95% CI: 1.19, 1.89. *p* = 0.0006) at 4 times/week of frequency, but the risk diminished with increasing stool frequency; e.g., adjusted HR = 1.26 (95% CI: 1.00, 1.57. *p* = 0.046) at 7 times/week. These individuals also showed lower risks (adjusted HRs < 1) of cancer mortality especially at higher stool frequencies of >7 times/week, but none of these were statistically significant. In contrast, a bell-shaped relationship was observed for CVD mortality with significantly elevated risks at stool frequencies of 6 to 12 times/week and the highest risk occurring at 9 times/week (adjusted HR = 2.26, 95% CI: 1.23, 4.14. *p* = 0.0083).

## 4. Discussion

The present study highlights the importance of and different conclusions from examining the joint association of stool frequency and stool consistency with mortality as opposed to only inspecting the individual associations of stool frequency or stool consistency on mortality like some prior analyses [21,22,24,34,35]. Without accounting for stool consistency, there seemed to be a higher risk of all-cause mortality but not cancer or CVD mortality at <7 times/week stool frequencies. Correspondingly, without accounting for stool frequency, there seemed to be a higher risk of all-cause and cancer mortality when comparing soft blob stools to normal smooth sausage-like stools. In fact, the results from the analysis of joint associations clarified the above two findings, revealing that the higher risk for soft stools occurred only at low stool frequencies but the conclusion applied to all-cause, cancer, and CVD mortality. Considering only individual associations also failed to capture the complexity of the different relationships between hard stools and mortality types. Infrequent hard stools were associated with higher risks of all-cause mortality, while regular or moderately frequent hard stools was associated with higher risks of CVD mortality. In contrast, hard stools did not contribute to higher risks of cancer mortality.

Constipation or lower stool frequency has been reported to have a higher risk of all-cause or CVD mortality [21,22,24,34,35]. The underlying mechanism could be dysbiosis, contributing to the pathogenesis of diverse illnesses [4], such as metabolic disorders [6,36,37,38], immune function [39], cardiovascular disease [40,41], and several cancers [2,42]. The altered gut microbiota has been cited to have a mediating role. Previous studies reported the correlations between stool frequency and gut microbiota, which interacted with host functions and involved in the associations between bowel disease and CVD through regulating immunology [5,22,43]. Abnormal stool status could reflect the dysbiosis of intestinal microbiota, which in turn affects human health. Gut dysbiosis in patients with constipation is mainly manifested by a decrease in species richness [44] (such as beneficial bacteria) and an increased concentration of methanogens [45], which are potentially pathogenic. As one of the clinical forms of dysbiosis, constipation is also related to altered gut metabolites (e.g., trimethylamine-N-oxide, etc.) [46]. Dysbiosis can participant in the pathogenesis of atherosclerosis through chronic inflammation and consequently lead to the higher incidence of CVD and all-cause mortality [24].

Our analyses indicate that infrequent soft or hard stools both contributed to higher all-cause mortality, but only infrequent soft stools were associated with higher CVD mortality. Therefore, these results not only agree with the previous reports but also extend them to delineate how stool consistency affects the association between constipation and cause-specific mortality. In contrast, the Nurses’ Health Study suggested that stool frequencies of more than once per day were associated with a 17% increase in cardiovascular mortality [4]. We also observed an increased risk in CVD mortality among participants with frequent hard stools, although its strength diminished for stool frequencies of twice or more per day.

Most of the prior research on the relationship between mortality and bowel health have focused on individual associations with stool frequency, constipation, or diarrhea. Moreover, analyses of stool frequency involved grouping the frequencies into different categories, resulting in precision loss and potential misclassification bias [47]. The various cutoffs used for discretization in different studies also hamper comparison. Our current study, however, directly models stool frequency as a quantitative variable, and can provide mortality estimates throughout the range of the variable. Its large study sample size rendered the possibility to jointly assess the associations of stool frequency together with stool consistency on mortality. More importantly, the use of a population-based survey sample enabled generalization to the U.S. adult population. To the best of our knowledge, this is the first nationally representative evaluation of bowel health on mortality for the U.S. population. Nevertheless, there are some limitations that warrant attention. First and foremost, the bowel health measures were self-reported. Research showed that people tend to exaggerate their defecation habits and overestimate the frequency of defecation [48,49,50]. Therefore, recall bias caused by self-report will affect the results. Secondly, the NHANES dataset only provided a baseline assessment so our study could not consider the temporal variability of the associations. Thirdly, we cannot exclude the possibility of unmeasured confounding in the associations. Fourth, we only considered fiber intake and adjusted in the models, there might be residual confounding from other dietary factors. Finally, the stool status (at recent 1 month) was self-reported by participants at baseline, which is a point estimation and cannot represent for the status during a median of 8 years long-term follow-up. Further longitudinal studies are warranted.

## 5. Conclusions

Stool consistency types and stool frequency are significant risk factors that are jointly associated with all-cause and cause-specific mortality, even after accounting for demographic and clinical variables and other behavioral factors such as alcohol and tobacco consumption, exercise, and dietary fiber intake. Specifically, infrequent soft stools were associated with a higher risk of all-cause, cancer, and CVD mortality, while regular or moderately frequent hard stools may contribute to a higher risk of CVD mortality. These findings suggest that self-monitoring of stool frequency and stool consistency may be a simple yet useful tool for informing about major chronic illnesses like cancer and CVD, and public health education and promotion should consider including similar bowel health evaluations.

## Figures and Tables

**Table 1:** Baseline characteristics by stool types among 14,574 participants from NHANES 2005–2010.

Characteristics	Total	Stool Consistency Types			*p*-Value
		Hard ^1^	Normal ^2^	Soft ^3^	
**No. of participants**	14,574	1128	11,152	2294	
**Bowel movements, %**					<0.0001
**1–2 times/week**	516 (3.3)	123 (11.0)	327 (2.7)	66 (2.8)	
**3–6 times/week**	1664 (12.1)	217 (21.1)	1272 (12.1)	175 (7.2)	
**7 times/week**	7386 (53.0)	487 (43.8)	5942 (55.6)	957 (42.7)	
**8–21 times/week**	4807 (30.4)	291 (23.4)	3519 (28.8)	997 (43.2)	
**>21 times/week**	201 (1.2)	10 (0.6)	92 (0.8)	99 (4.1)	
**Age, years**	46.7 ± 0.3	45.7 ± 0.5	46.5 ± 0.4	48.3 ± 0.5	<0.0001
**Females%**	7402 (51.1)	777 (71.7)	5353 (48.6)	1272 (55.0)	<0.0001
**Race/Ethnicity%**					<0.0001
**non-Hispanic Black**	2890 (10.9)	257 (14.6)	2175 (10.4)	458 (12.0)	
**non-Hispanic White**	7184 (71.1)	488 (64.8)	5700 (72.6)	996 (65.6)	
**Mexican American**	2677 (8.2)	216 (9.2)	1948 (7.6)	513 (10.9)	
**Others**	1823 (9.8)	167 (11.4)	1329 (9.4)	327 (11.5)	
**Smoking status%**					0.0001
**Never**	7651 (52.8)	670 (59.1)	5846 (52.9)	1135 (48.8)	
**Former smoker**	3703 (24.9)	242 (20.9)	2859 (25.1)	602 (25.8)	
**Current smoker**	3215 (22.3)	216 (20.0)	2443 (21.9)	556 (25.4)	
**Current drinker%**	10,414 (75.9)	678 (65.1)	8164 (77.3)	1572 (73.7)	
**Recreational physical activity%**					<0.0001
**Low or never**	5720 (47.8)	495 (53.1)	4130 (45.6)	1095 (56.2)	
**Moderate**	2657 (28.4)	183 (26.5)	2069 (29.3)	405 (24.4)	
**Vigorous**	1965 (23.8)	131 (20.4)	1582 (25.1)	252 (19.3)	
**BMI kg/m^2^**	28.7 ± 0.1	27.8 ± 0.3	28.5 ± 0.1	30.2 ± 0.2	<0.0001
**Underweight**	219 (1.6)	24 (1.9)	167 (1.7)	28 (1.1)	<0.0001
**Normal weight**	3919 (29.8)	361 (36.0)	3075 (30.2)	483 (24.6)	
**Overweight**	4965 (33.7)	371 (33.2)	3857 (34.4)	737 (29.9)	
**Obesity**	5336 (34.9)	360 (28.8)	3958 (33.8)	1018 (44.4)	
Dietary fiber g *	16.3 ± 0.2	14.1 ± 0.3	16.6 ± 0.2	16.8 ± 0.2	<0.0001
**Annual family income%**					<0.0001
**≤$19,999**	3491 (17.3)	322 (22.4)	2529 (16.4)	640 (19.8)	
**$20,000–$34,999**	3442 (20.2)	289 (24.3)	2578 (19.6)	575 (21.9)	
**$35,000–$74,999**	4069 (31.5)	288 (29.8)	3173 (31.7)	608 (30.9)	
**≥$75000**	3051 (31.0)	176 (23.5)	2487 (32.2)	388 (27.4)	
**History of chronic disease%**	6509 (40.6)	462 (40.3)	4860 (39.4)	1187 (47.4)	<0.0001

^1^. Hard includes Type 1 (“Separate hard lumps, like nuts”) and Type 2 (“Like a sausage but lumpy”); ^2^. Normal includes Type 3 (A sausage shape with cracks in the surface) and Type 4 (“Like a sausage or snake, smooth and soft”); ^3^. Soft includes Type 5 (“Soft blobs with clear-cut edges”), Type 6 (“Fluffy pieces with ragged edges, a mushy stool”) and Type 7 (“Watery, no solid pieces”). * Means ± SEs were calculated for continuous variables.

**Table 2:** Baseline characteristics by stool frequency among the population of NHANES 2005-2010.

Characteristics	Stool Frequency					*p*-Value
	1–2 Times/Week	3–6 Times /Week	7 Times/Week	8–21 Times/Week	>21 Times/Week	
**Number of participants**	516	1664	7386	4807	201	
**Age, years**	43.0 ± 0.9	44.6 ± 0.5	47.6 ± 0.4	46.3 ± 0.4	49.7 ± 1.2	<0.0001
**Female, n (%)**	401 (83.4)	1081 (67.7)	3826 (52.1)	1997 (39.5)	97 (44.7)	<0.0001
**Ethnicity, n (%)**						<0.0001
**non-Hispanic Black**	172 (21.5)	383 (12.8)	1224 (8.4)	1076 (13.5)	35 (10.9)	
**non-Hispanic White**	235 (65.3)	895 (73.8)	4038 (75.1)	1921 (63.7)	95 (67.8)	
**Mexican American**	57 (5.6)	197 (5.0)	1186 (6.7)	1186 (12.3)	51 (10.9)	
**Others**	52 (7.6)	189 (8.6)	938 (9.8)	624 (10.5)	20 (10.4)	
**Smoking status, n (%)**						0.0001
**Never**	273 (52.5)	935 (56.8)	3830 (52.1)	2508 (52.4)	105 (52.6)	
**Former smoker**	100 (19.0)	367 (21.0)	1972 (26.1)	1211 (25.1)	53 (25.7)	
**Current smoker**	143 (28.5)	362 (22.2)	1582 (21.8)	1085 (22.5)	43 (21.7)	
**Current drinker, n (%)**	319 (64.0)	1159 (74.3)	5260 (75.8)	3536 (78.2)	140 (70.5)	<0.0001
**Recreational physical activity, n (%)**						0.0034
**Low or never**	226 (57.2)	674 (48.7)	2826 (47.0)	1915 (47.8)	79 (45.8)	
**Moderate**	87 (29.1)	287 (29.1)	1391 (29.3)	849 (26.3)	43 (30.0)	
**Vigorous**	47 (13.7)	198 (22.2)	960 (23.7)	740 (25.9)	20 (24.2)	
**BMI, kg/m^2^**	28.0 ± 0.3	27.7 ± 0.2	28.3 ± 0.1	29.8 ± 0.2	30.7 ± 0.5	<0.0001
**Underweight, n (%)**	8 (1.7)	35 (2.3)	126 (1.8)	47 (0.9)	3 (1.4)	<0.0001
**Normal weight, n (%)**	170 (35.7)	551 (36.4)	2118 (31.3)	1047 (24.3)	33 (18.5)	
**Overweight, n (%)**	162 (31.3)	538 (31.9)	2574 (34.2)	1624 (33.7)	67 (35.2)	
**Obesity, n (%)**	170 (31.3)	517 (29.4)	2498 (32.7)	2056 (41.1)	95 (44.9)	
Dietary fiber, g *	12.4 ± 0.4	14.7 ± 0.3	16.1 ± 0.2	17.6 ± 0.3	16.9 ± 0.8	<0.0001
**Annual family income, n (%)**						<0.0001
**≤$19,999**	181 (29.8)	389 (17.7)	1690 (16.1)	1166 (17.7)	65 (24.2)	
**$20,000–$34,999**	117 (21.7)	393 (19.0)	1723 (20.1)	1167 (21.1)	42 (17.6)	
**$35,000–$74,999**	123 (27.6)	486 (32.9)	2089 (31.7)	1320 (31.0)	51 (29.2)	
**≥$75,000**	73 (20.8)	354 (30.3)	1638 (32.1)	949 (30.3)	37 (28.9)	
**History of chronic disease, n (%)**	223 (41.5)	695 (37.0)	3266 (39.8)	2200 (42.6)	125 (59.3)	<0.0001

* Means (SEs) were calculated for continuous variables.

**Table 3:** Associations between the stool consistency and all-cause, cancer-, and CVD-specific mortality.

Stool Consistency	Proportion of Deaths	Mortality Rate (per 100,000 Person Years)	HR (95% CI)
**All mortality**			
**Separate hard lumps, like nuts**	40/319	1302	1.36 (0.99, 1.88)
**Like a sausage but lumpy**	93/809	990	1.23 (0.98, 1.56)
**A sausage shape with cracks in the surface**	313/3607	762	1.13 (0.94, 1.36)
**Like a sausage or snake, smooth and soft**	769/7545	922	Ref.
**Soft blobs with clear-cut edges**	128/1162	1189	1.48 ** (1.17, 1.86)
**Fluffy pieces with ragged edges, a mushy stool**	131/983	1323	1.20 (0.88, 1.64)
**Watery, no solid pieces**	28/149	2452	1.41 (0.86, 2.33)
**Cancer mortality**			
**Separate hard lumps, like nuts**	6/319	252	1.25 (0.39, 3.95)
**Like a sausage but lumpy**	13/809	106	0.57 (0.30, 1.07)
**A sausage shape with cracks in the surface**	78/3607	191	1.21 (0.88, 1.66)
**Like a sausage or snake, smooth and soft**	187/7545	219	Ref.
**Soft blobs with clear-cut edges**	31/1162	310	1.60 * (1.02,2.50)
**Mushy stool, or watery**	42/1132	333	1.25 (0.69, 2.29)
**CVD mortality**			
**Separate hard lumps, like nuts**	9/319	283	1.80 (0.84, 3.88)
**Like a sausage but lumpy**	19/809	191	1.45 (0.87, 2.41)
**A sausage shape with cracks in the surface**	62/3607	136	1.04 (0.76, 1.43)
**Like a sausage or snake, smooth and soft**	145/7545	171	Ref.
**Soft blobs with clear-cut edges**	26/1162	218	1.48 (0.86, 2.56)
**Mushy stool, or watery**	23/1132	241	1.19 (0.70, 2.00)

The Cox regression model adjusted for age, sex, race/ethnicity, BMI, physical activity, smoking status, dietary fiber intake, alcohol consumption, and annual family income. * *p*-value < 0.05, ** *p*-value < 0.01. Type 7 (Watery, no solid pieces) was incorporated into type 6 (Fluffy pieces with ragged edges, a mushy stool) for analysis since it had less than 5 deaths.

**Table 4:** Joint associations of the stool frequency and stool consistency with all-cause, cancer, and CVD mortality.

Outcome	Joint Association/Type and Frequency	HR *	*p*-Value
**All-cause**	Hard stool at 4 times/week	1.50 (1.19, 1.89)	0.0006
	Hard stool at 7 times/week	1.26 (1.00, 1.57)	0.0456
	Hard stool at 14 times/week	0.97 (0.75, 1.26)	0.8249
	Hard stool at 21 times/week	0.79 (0.44, 1.43)	0.4348
	Soft stool at 4 times/week	1.78 (1.17, 2.73)	0.0076
	Soft stool at 7 times/week	1.32 (1.06, 1.64)	0.0120
	Soft stool at 14 times/week	1.17 (0.94, 1.46)	0.1567
	Soft stool at 21 times/week	1.27 (0.98, 1.64)	0.0696
**Cancer**	Hard stool at 4 times/week	0.85 (0.48, 1.51)	0.5709
	Hard stool at 7 times/week	0.70 (0.38, 1.28)	0.2478
	Hard stool at 14 times/week	0.55 (0.17, 1.74)	0.3086
	Hard stool at 21 times/week	0.46 (0.05, 4.27)	0.4972
	Soft stool at 4 times/week	2.42 (1.31, 4.47)	0.0048
	Soft stool at 7 times/week	1.30 (0.86, 1.95)	0.2078
	Soft stool at 14 times/week	0.94 (0.59, 1.48)	0.7846
	Soft stool at 21 times/week	1.00 (0.62, 1.61)	0.9911
**CVD**	Hard stool at 4 times/week	1.23 (0.56, 2.69)	0.6123
	Hard stool at 7 times/week	2.05 (1.16, 3.64)	0.0137
	Hard stool at 14 times/week	1.50 (0.77, 2.90)	0.2296
	Hard stool at 21 times/week	0.64 (0.19, 2.19)	0.4794
	Soft stool at 4 times/week	2.27 (1.12, 4.60)	0.0231
	Soft stool at 7 times/week	1.42 (0.91, 2.21)	0.1194
	Soft stool at 14 times/week	0.95 (0.54, 1.67)	0.8630
	Soft stool at 21 times/week	0.81 (0.41, 1.62)	0.5528

The Cox regression model adjusted for age, sex, race/ethnicity, BMI, physical activity, smoking status, dietary fiber intake, alcohol consumption, and annual family income. * HRs are calculated in comparison to the reference of normal stool at 7 times/week and are presented at stool frequencies that are ≥5% marginally.

## Data Availability

The datasets generated and analyzed during the current study are available on the NHANES website: https://www.cdc.gov/nchs/nhanes/index.htm (accessed on 25 September 2020).

## Abbreviations

NHANES	National Health and Nutrition Examination Survey
HR	hazard ratio
CVD	cardiovascular disease
ICD	international classification of disease
BMI	body mass index
ANOVA	analysis of variance
CI	confidence intervals
